# Detection of genome-edited mutant clones by a simple competition-based PCR method

**DOI:** 10.1371/journal.pone.0179165

**Published:** 2017-06-06

**Authors:** Takeshi Harayama, Howard Riezman

**Affiliations:** Department of Biochemistry and NCCR Chemical Biology, Sciences II, University of Geneva, Geneva, Switzerland; Osaka University, JAPAN

## Abstract

Genome editing by the CRISPR/Cas9 (clustered regularly interspaced short palindromic repeats / CRISPR-associated protein 9) system is a revolutionary strategy to study gene functions. Since the efficiency of gene disruption in cell culture does not reach 100% typically, cloning of mutant cells is often performed to obtain fully mutated cells. Therefore, a method to discriminate accurately mutated clones easily and quickly is crucial to accelerate the research using CRISPR/Cas9. Here, we show that knockout cells can be discriminated by a competition-based PCR, using a mixture of three primers, among which one primer overlaps with the Cas9 cleavage site. Together, we show how to optimize primer design in order to improve the effectiveness of the discrimination. Finally, we applied this method to show that mutations conferring drug resistance can be detected with high accuracy. The provided method is easy to perform and requires only basic laboratory equipment, making it suitable for almost all laboratories.

## Introduction

Recently, genome editing by the CRISPR/Cas9 system has became a widely used strategy to study gene functions in cells and *in vivo* [1]. In the widely-used CRISPR/Cas9 system derived from *Streptococcus pyogenes*, the Cas9 nuclease creates a double strand break in the genome at a site complementary to the guide RNA, which is then repaired by various repair machineries of the cell. This often leads to the introduction of indels, resulting in an efficient loss of function of gene. However, even with the most efficient delivery strategies, gene disruption does not reach 100% efficiency in cell culture [2]. For this reason, researchers have often performed isolation of mutant clones before doing their assay of interest [3]. Therefore, a strategy to identify mutant clones quickly and accurately is very important. The indels caused by CRISPR/Cas9 editing are often small, thus standard PCR with genomic DNA (gDNA) is not useful to detect the mutants since the amplicon sizes are not different enough. The usage of Cas9 nuclease with two guide RNAs targeting relatively distant sites enables an easy discrimination by inducing a bigger deletion detectable by PCR [4] (not to be confounded with the double-nickase strategy where the offset should be small enough to get a double strand break [5]). However, with this strategy only clones having the large deletion in all alleles are discriminated, and the potentially useful mutant clones having only small indels are ignored, reducing the success rate of obtaining mutants. Also, this strategy doubles the effort of plasmid preparation as well as the chance of having off-target effects.

Multiple methods to identify CRISPR/Cas9-induced indels have been published. A strategy that is often used is the mismatch cleavage assay [3]. This method uses enzymes that cleave DNA heteroduplexes at mismatch sites. Although being easy to perform, this method does not discriminate wild type clones and mutant clones harbouring identical mutations in both alleles. Also, heterozygous mutants are not discriminated from homozygous mutants with different mutations between alleles. These issues can be avoided by mixing wild type PCR amplicon with the amplicons of the analysed samples. However, this adds another step for the experiment, and DNA concentrations of PCR products have to be quantified in order to have a correct ratio of mixed amplicons, since this affects the cleavage efficiency. An ideal method should discriminate completely mutated cells from the others without the need of too many steps, while being cost effective, and not requiring special laboratory equipment. In case of difficult-to-transfect cells or when doing multiplex gene disruption, hundreds of clones might have to be analysed. Therefore, it is also desirable to reduce any steps like template DNA quantification and PCR product purification.

None of the existing methods fulfil all the above requirements. For example, high-resolution melting analysis is easy and quick, but requires specific instruments [6]. Also, since different type of indels will affect the melting behaviour of PCR products, unambiguous identification of homozygous mutants might be difficult. Capillary electrophoresis of PCR products enables accurate detection of indels, but requires specific instruments [7]. Quantitative PCR efficiently identifies mutations, but the highly quantitative nature of this method gives rise to the need to quantify and normalize template DNA amount [8]. Heteroduplex mobility assay is simple to perform, but has similar drawbacks to mismatch cleavage assays when applied to clone analysis [9]. Restriction fragment length polymorphism and RNA-guided engineered nuclease restriction fragment length polymorphism are almost ideal [10]. However, the former requires a convenient restriction site at the cleavage site, and for the latter one needs to generate *in vitro* the nuclease complex used for restriction analysis, which might be laborious. In addition, enzyme restriction is another step added to PCR, which one might want to avoid if possible. Most of the methods stated above have the benefit of being able to estimate the mutation efficiency (with more or less accuracy) in bulk cells. This degree of quantitation is not required when the only requirement is to detect fully mutated clones. Thus, we wanted to establish a method that is easy to perform without the need to normalize template DNA amount, and requiring only minimal laboratory instruments, even if it might be less quantitative than the ones stated above. Here, we show that when primers are appropriately designed, a simple PCR using a set of primers flanking the target site of interest, together with an additional one overlapping with the site cleaved by Cas9, enables the identification of homozygous mutant clones.

## Materials and methods

### Cell culture

McA-RH7777 cells and HeLa MZ cells were cultured in Dulbecco’s Modified Eagle Medium (DMEM high glucose, GlutaMAX, pyruvate), supplemented with 10% fetal bovine serum and 100 U/mL penicillin-streptomycin (all from Thermo Fisher Scientific, Waltham, MA, USA).

### Plasmid construction for genome editing

Plasmid backbones for CRISPR/Cas9 experiments were either pX330 [3], which contains expression cassettes for both Cas9 and single guide RNAs (sgRNAs), or an in-house constructed plasmid (pUC-U6-sg) for expressing only sgRNAs. The latter was constructed by incorporating the sgRNA expression cassette from pX330 into a pUC19 backbone. The insert and the pUC19 backbone were amplified by PCR using PrimeSTAR GXL DNA Polymerase (TAKARA Clonthech, Otsu, Shiga, Japan) with the primers listed in S1 Table, and assembled using the Gibson assembly system (New England Biolabs, Ipswich, MA, USA). Target sites for genome editing (S2 Table) were selected using either the Zhang laboratory algorithm [3] (http://crispr.mit.edu) or CRISPOR [11] (http://crispor.tefor.net), choosing those having low off-target scores and high on-target scores [12]. The pairs of oligo DNA were synthesized at Microsynth AG (Balgach, St. Gallen, Switzerland). Plasmids were constructed by Golden Gate assembly, based on the Yamamoto laboratory protocol [13] with slight modifications. Restriction and ligation was performed in a single tube using FastDigest Bpi I (Thermo Fisher Scientific) and quick ligase (New England Biolabs, Ipswich, MA, USA) in quick ligase buffer. Tubes were incubated at 37°C and 25°C for 5 minutes each, and this cycle was repeated three times. Then, an additional Bpi I digestion was done for 1 hour. Products were transformed into STBL3 chemical competent cells (Thermo Fisher Scientific), plasmids were sequence-verified by Sanger sequencing by Fasteris SA (Plan-les-Ouates, Geneva, Switzerland), and obtained in large scale using either QIAFilter plasmid midiprep kit (QIAGEN, Hilden, Germany) or PureLink HiPure midiprep kit (Thermo Fisher Scientific).

### Genome editing in cultured cells

Genome editing in McA-RH7777 cells was performed based on a previously described Hprt co-targeting strategy [14], which was optimized for our cell lines. In this method, mutant cells are strongly enriched by selecting cells having a mutation in the co-targeted Hprt1 gene, which confers resistance to 6-thioguanine (6-TG, Sigma-Aldrich, St. Louis, MO, USA). Transfection of plasmids (450 ng of pX330-based plasmid for the target gene, and 50 ng of pUC-U6-sg-based plasmid for rat Hprt1 gene) was done using lipofectamine 3000 (Thermo Fisher Scientific), by reverse transfection; the mixed transfection reagents were put in an empty 24 well plate, and 100,000 cells were seeded on them in a volume of 1 mL. Cells were maintained in culture with occasional passages. Nine days post-transfection, cells were detached and diluted in a medium containing 4 μg/mL 6-TG. Selection was done for one week, with a medium change at day 4. To isolate triple knockout clones, cells were transfected with equal amounts of the three pX330-based plasmids to target Sgpl1, Sgpp1, and Sgpp2, and subjected to limiting dilution 5 days post-transfection on a type I collagen (Sigma-Aldrich)-coated 96 well plate. Conditioned medium was added at 20% to promote the growth of clonal populations. Genome editing in HeLa MZ cells was done without co-targeting (empty pX330 plus pUC-U6-sg-based plasmid for human HPRT1 gene), using the same transfection method. Clones of HeLa MZ cells were obtained by limiting dilution into 96 well plates. To analyse polyclonal HPRT1-mutated HeLa MZ cells, the transfectants were selected with 4 μg/mL 6-TG 5 days post-transfection.

### Primer design

Primer sequences for the amplification of genomic DNA were designed using primer-BLAST [15] (http://www.ncbi.nlm.nih.gov/tools/primer-blast/). Melting temperature was calculated using the same program. The primers were typically designed to amplify 700 to 900 bp, with the Cas9-cleaved site located between 150 to 300 bp from one end. For competition-based PCR, the inner primers were designed only after amplification of the outer amplicon was confirmed. Except for the initial experiments during optimization, the inner primers were designed to have a melting temperature not exceeding 60°C, with the 3’ end extending 3 bases beyond the cleavage site. If this design resulted a primer shorter than a 16-mer, the primer was designed as a 16-mer regardless of its melting temperature. Then, the outer primer having the same orientation with the inner one was redesigned (when required) to have a lower melting temperature than it. Details are provided in S1 Protocol.

### Isolation of genome DNA and confirmation of mutation rates

Genomic DNA was isolated as following. Cells were lysed in lysis buffer (20 mM Tris-HCl, pH 8.0, 1 mM EDTA, 0.67% (w/v) SDS, and 124 μg/mL proteinase K (Roche Life Science, Rotkreuz, Zug, Switzerland)) at 55°C for at least 4 hours. DNA was precipitated with isopropanol, pellets were washed with 70% ethanol, and were dissolved in TE buffer (20 mM Tris-HCl, pH 8.0, 1 mM EDTA). TIDE (Tracking of Indels by DEcomposition) [16] analysis was performed to confirm that samples used in the optimization steps are mutants. For this, target regions were amplified by PCR (primers are listed in S1 Table), amplicons were treated with Exonuclease I and FastAP alkaline phosphatase (both from Thermo Fisher Scientific), followed by heat denaturation, and directly used for Sanger sequencing. Competition-based PCR was performed using a mixture of 3 primers at 0.2 μM each. PCR reactions were carried out using ExTaq polymerase (Takara Clontech) or PrimeSTAR GXL polymerase. For ExTaq reactions, 5% (v/v) DMSO was added. PCR reactions with the following temperature cycles were performed using a T Professional TRIO Thermocycler (Biometra, Göttingen, Germany): 30 seconds at 94°C, 30 seconds at 60°C, and 50 seconds at 72°C (total 35 cycles). For PrimeSTAR GXL reactions, the conditions were: 10 seconds at 98°C, 15 seconds at 60°C, and 50 seconds at 68°C (35 cycles). PCR products were analysed by agarose gel electrophoresis, typically using 1.5% (w/v) gels in TBE buffer (89 mM Tris-borate, 2 mM EDTA). Bands were visualized using EZ-VISION DNA dye (amresco, Solon, OH, USA) or ethidium bromide (Sigma-Aldrich). Primers are listed in S3 Table.

### Image processing and quantification of band signals

For clarity on printed pages, signals of some images were adjusted using photoshop (Adobe, San Jose, CA, USA), while maintaining the linearity of signals. For quantification of signals, images without adjustment were used. The digital gel images were analysed using ImageJ. We obtained images using different exposure times, and did quantification with those that did not have saturating signals.

### Analysis of 6-TG sensitivity

Individual HeLa MZ clones were placed in different wells of a 96 well plate with approximately 20% confluency, and incubated with 6 μg/mL 6-TG (day 0). Medium change (still containing 6-TG) was done at day 4, and cells were fixed with methanol at day 6. Cells were stained with 0.5% crystal violet (in 20% methanol) for visualization.

### Analysis of mutant clones by sequencing

To analyse genome sequencing results of clones that were selected by competition-based PCR, TIDE analysis was done for each locus. When TIDE analysis gave sufficient explanation for the mixed sequencing chromatograms, it was concluded that the clone contained the indels proposed by the algorithm. When an indel larger than 50 bases was present, this allele could not be calculated by TIDE, so chromatogram deconvolution was done manually. Control and mutant chromatograms were compared by eye, and the aberrant sequence was estimated from their difference, which was matched with downstream sequence in case of large deletions. If a large stretch of aberrant sequence was found to be not corresponding to any sequence of the locus, this was interpreted as an insertion of a relatively random sequence.

When different alleles of mutant clones had to be analysed, target regions were amplified by PCR using PrimeSTAR GXL (primers at S1 Table), amplicons were cloned using Zero Blunt TOPO PCR Cloning Kit (Thermo Fisher Scientific), and transformed into STBL3 chemical competent cells. Bacterial colonies containing plasmids with inserts were selected by colony-direct PCR, and these PCR products were used for direct Sanger sequencing after Exonuclease I and FastAP alkaline phosphatase treatment as written above.

## Results

### Competition-based PCR detects mutant cells

A previous study showed that quantitative PCR with one of the primers overlapping with the Cas9 cleavage site enables the detection of mutations in zebrafish [8]. In the same study, endpoint PCR was used only to detect mutant fragments after isolation into plasmids. This motivated us to develop an endpoint PCR-based method to detect mutant clones of cultured cells, without the need to isolate fragments into plasmids. To optimize such a method, we used gDNA from mutant cells that were established for another unpublished study. Since this manuscript focuses on the detection strategy, details about the generation and characterization of these mutant cells will be described elsewhere. In this study, the PCR strategy is designed to detect mutations generated by wild type *Streptococcus pyogenes* Cas9 nuclease (hereafter simply described as Cas9). The potential use of this strategy to detect mutations induced by other genome editing methods will be addressed in the Discussion.

**Fig 1:**
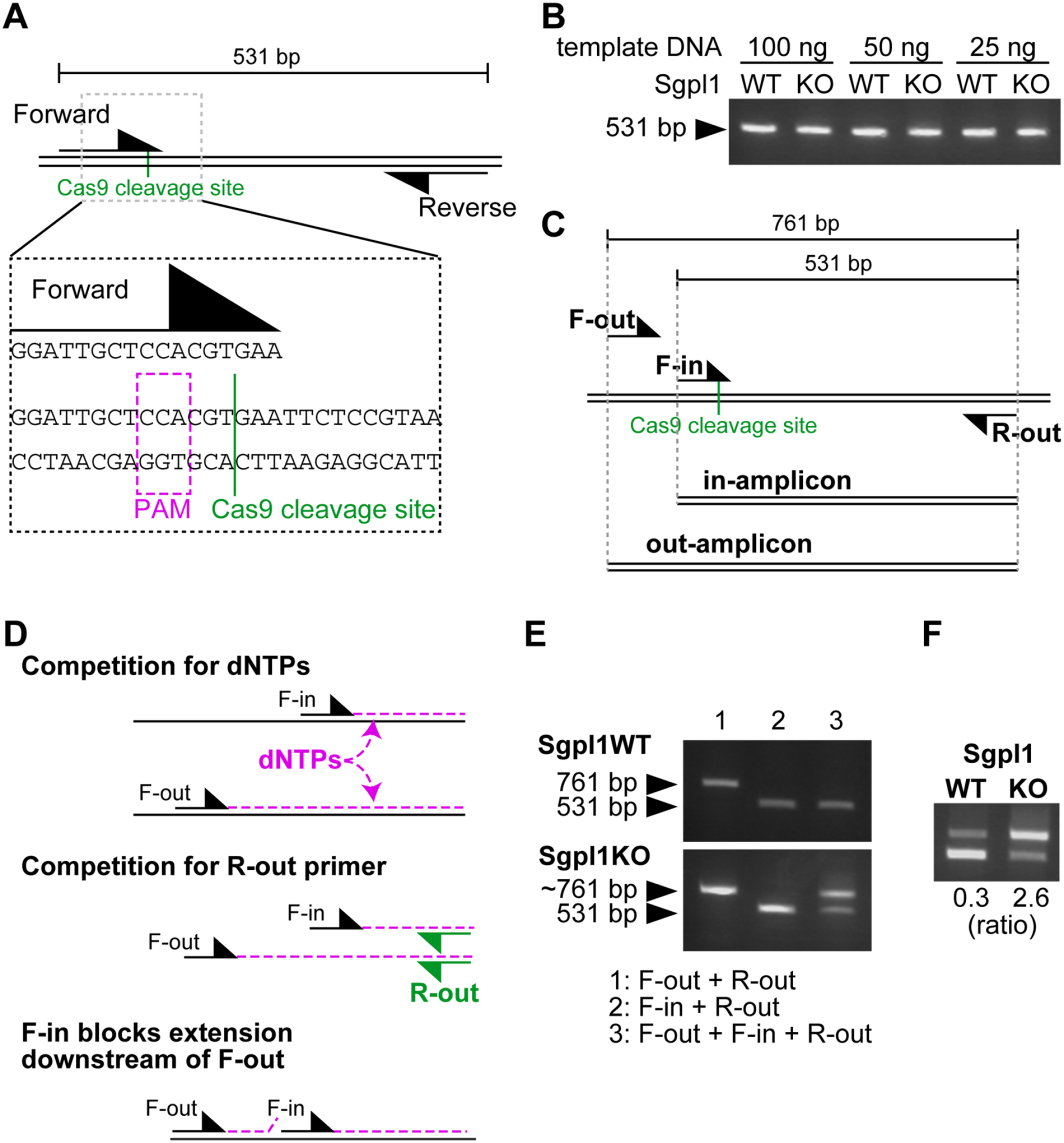
Competition between amplicons enables the discrimination of mutant cells. (A) Design of the PCR primers to test whether Sgpl1-mutant cells can be discriminated by conventional endpoint PCR. The 3' end of the forward primer is zoomed to show its location relative to the Cas9 cleavage site. (B) Conventional endpoint PCR with the primer design shown in (A) was performed using wild type or mutant gDNA. The levels of PCR products did not change depending on genotypes or template DNA amount. (C) Design of competition-based PCR (cbPCR) and nomenclature of primers and amplicons. F-in and R-out are the same primers as forward and reverse primers in (A), respectively. (D) Basis of competition between amplicons that occur during cbPCR. (E) PCR was performed with the indicated primer sets, using gDNA from wild type or Sgpl1-mutant cells. The pattern of competition between cbPCR amplicons differed between wild type and mutant gDNA. (F) In contrast to conventional endpoint PCR, cbPCR enables the discrimination of wild type and mutant gDNA. The ratios between out- and in-amplicons are indicated. WT: wild type, KO: knockout (mutant).

**Fig 2:**
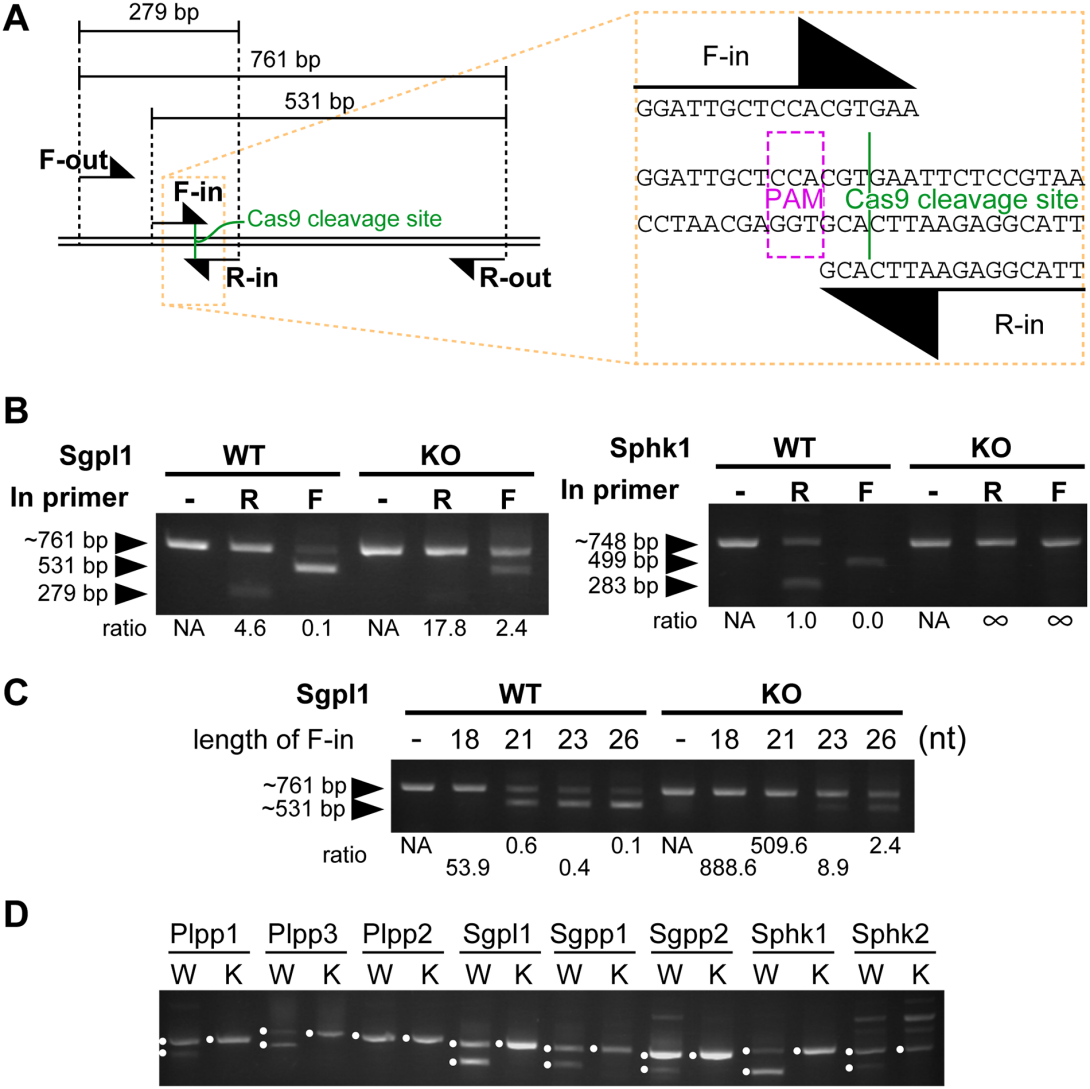
Optimization of cbPCR. (A) Nomenclature of the primers based on their orientation, using Sgpl1 locus as an example. The Cas9 cleavage site is zoomed in to show where the inner primers bind. (B) PCR was performed with or without the addition of the indicated inner primer. The ratios between out- and in-amplicons were calculated. The orientation of the inner primer affected the degree of competition between amplicons. See the changes in the 761 bp- or 748 bp-outer amplicons. The same experiment is performed in two different loci, namely Sgpl1 and Sphk1. (C) PCR was performed with the addition of inner primers of different lengths. The ratios between out- and in-amplicons were calculated. The length of the inner primer affected the degree of competition between amplicons (as seen by the decrease of the 761 bp-outer amplicon) and the tolerance to the mutations (as seen by the appearance of the ~531 bp-inner amplicon when using mutant DNA). (D) Mutants can be discriminated by cbPCR in most of the targets with a common primer design. Bands with expected sizes are marked with dots. The other bands were ignored as being either nonspecific or heteroduplexes. WT or W: wild type, KO or K: knockout (mutant).

We used McA-RH7777 cells that have mutations in Sgpl1 gene for initial optimization. First, we performed a standard endpoint PCR with one primer overlapping with the Cas9 cleavage site, placing the cleavage site between the third and fourth bases from the 3' end of the primer (Fig 1A). As can be expected, the amount of PCR products did not differ when using gDNA from wild type or mutant cells, nor did it when using different template DNA amounts (Fig 1B). Thus, as is well known, amplification reaches a plateau in conventional end point PCR, and even if the affinity of the primer with template DNA from mutant cells is reduced, the amount of final products is unchanged [17]. This shows that mutants cannot be discriminated using this design of primers, unless using quantitative PCR as in the previous study [8]. Next, we designed a PCR strategy where one pair of primers is designed to flank the Cas9 cleavage site (the outer primers), and another primer (the inner primer) overlaps with the cleavage site (Fig 1C). Among the outer primers, the one proximal to the Cas9 cleavage site will be called the “F-out” primer, and the other one the “R-out” primer. The inner primer will be called “F-in” when it directs to the same direction as F-out, and “R-in” in the other case (Fig 1C, see also Fig 2A for the R-in orientation). The amplicon of outer primers will be called “out-amplicon”, and the one from the inner primer and the corresponding outer primer will be called “in-amplicon” (Fig 1C). Under such a PCR condition, competition between amplicons should occur (Fig 1D). First, since the amount of substrates (dNTPs and primers) is not infinite, the amplification of one amplicon reduces the amount of substrates that can be used for the other. Second, if the inner primer is present on a template DNA, the elongation from the outer primer that is located upstream will be blocked (unless using a polymerase with strand-displacing activity). Thus, when an amplicon is strongly amplified, the other one is reduced. The fact that out-amplicons can be used as a template for PCR of in-amplicon, but not the inverse, makes this competition even more complex (not depicted in the Figure). When gDNA from a mutant is utilized, the efficiency of inner primer binding should be lower, shifting the balance of amplification toward the out-amplicon. We thought that this design would lead to a different ratio between out- and in-amplicons when using mutant gDNA, enabling their discrimination from wild type. This design was inspired by the fact that before the appearance of real-time PCR, quantitative PCR was done based on competition of primers caused by the addition of a competitor DNA [17, 18]. The difference of the current design is that it does not require the preparation of a competitor DNA.

Based on this design, we tested whether competition between individual amplicons indeed occurs. We did PCR of the Sgpl1 locus from wild type gDNA using individual primer pairs (F-out and R-out or F-in and R-out), or with the three primers mixed (Fig 1E, upper). The signal of the out-amplicon was strongly inhibited when the inner primer was present (lanes 1 versus 3). On the other hand, the signal of the in-amplicon was unaffected by the presence of F-out primer (lanes 2 versus 3). We did the same experiment using genomic DNA from Sgpl1 mutant cells. This time, the signals of both of the amplicons were weaker when the three primers were used than when doing PCR individually (Fig 1E, bottom). Thus, as assumed, each amplicon has a negative effect on the other. In wild type cells, the out-amplicon was so strongly inhibited that it could not affect the amount of the in-amplicon. The balance of inhibition was different in mutant cells due to the lower affinity of the inner primer. Therefore, wild type and mutant DNA could be discriminated by calculating the ratio of out- and in-amplicon when doing PCR with the three primers (Fig 1F). We will call this PCR method “competition-based PCR (cbPCR)”.

### Optimization of cbPCR

Having shown that cbPCR can discriminate mutant DNA from wild type, we next investigated the factors that affect the efficiency of this method in order to reduce the chance of potential identification errors. This was important since CRISPR/Cas9 leads to various indel sizes [19], thus some might be more or less easily discriminated (due to differences in inner primer affinity), and we thought that optimization was required to enable discrimination of all of them from wild type. We first tested whether there is an optimal orientation of the inner primers (Fig 2A). Based on the nomenclature defined above, the F-in primer provides a larger in-amplicon, which utilizes more dNTPs. We did cbPCR using F-in or R-in as the inner primer (Fig 2B). The use of F-in led to a stronger inhibition of out-amplicons than when R-in was used. Both primers enabled the discrimination of mutant and wild type DNA using the ratio of out- and in-amplicons, but the difference was bigger when F-in was used. This enabled a better genotype discrimination due to a stronger inhibition of out-amplicon when using wild type DNA. The same pattern was seen when another gene, Sphk1, was tested (Fig 2B). In this case, in-amplicons were absent when cbPCR was done with mutant DNA regardless of inner primer orientation, but the use of F-in led to a stronger inhibition of out-amplicon when using wild type DNA. Thus, when primer design allows it, the inner primer should be designed in the direction of F-in.

We next tested the effect of F-in primer length. A longer F-in primer might increase its affinity with template DNA, leading to a stronger inhibition of out-amplicon when using wild type DNA, thus improving mutant discrimination. However, a longer primer might also increase the tolerance to mutation in the template, reducing mutant discrimination. Therefore, it was difficult to predict whether the F-in primer should be long or short. We did cbPCR using F-in primers of varying lengths (Fig 2C). As expected, a longer F-in primer leads to a smaller out- to in-amplicon ratio in wild type DNA, but at the same time increased the tolerance for mutations (see the appearance of in-amplicons in PCR products of mutant gDNA). Therefore, the best discrimination was achieved when the F-in primer was short, as long as it enabled efficient detection of in-amplicon in wild type DNA (21 mer in Fig 2C). In our PCR conditions, we usually achieved this condition when the F-in primer was designed to be the longest while not exceeding a melting temperature of 60°C. We also routinely designed the F-out primer with a melting temperature lower than the F-in primer, in order to increase the inhibition of out-amplicon when doing cbPCR with wild type DNA. We used this primer design as a default. We next tested whether by using this default setting, we could discriminate mutants of different genes by cbPCR. For this, we used gDNA from 8 mutant cell lines that were generated a priori. Without further optimization of primer design, we could clearly discriminate 7 out of 8 mutant lines from wild type using cbPCR, by the absence of in-amplicons (Fig 2D). The exception was the Plpp2 gene, where the in-amplicon was also absent in cbPCR product of wild type gDNA. Except for this, using the default primer settings, most of mutants could be discriminated by cbPCR.

### Guidelines for primer refinement

**Fig 3:**
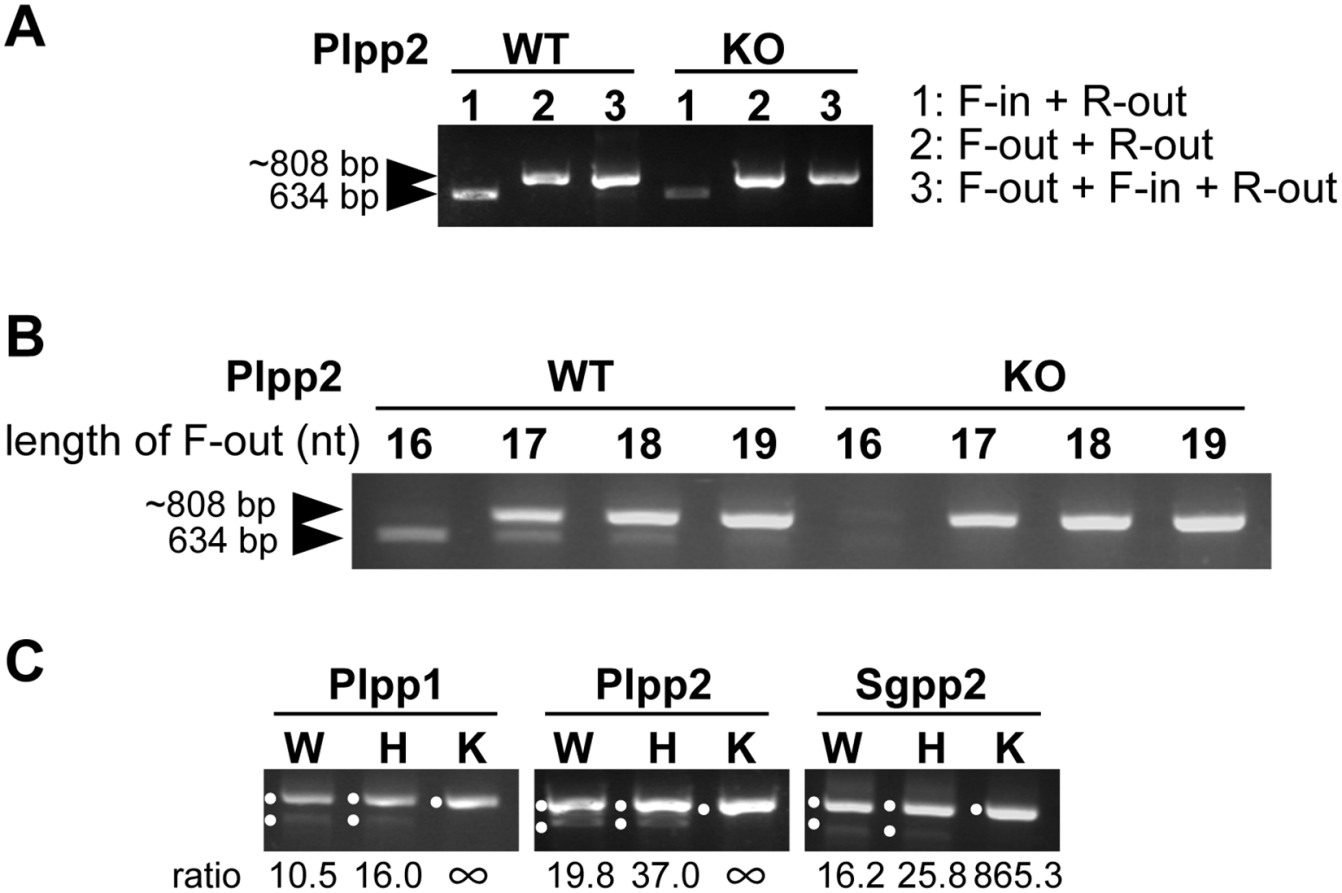
Refinement of cbPCR. (A) PCR of Plpp2 locus was performed using the indicated primer sets. When F-out was absent, Plpp2 inner amplicon was detected in wild type DNA (lane 1), but was barely detected when doing cbPCR (lane 3). (B) Decreasing F-out primer length enabled detection of inner amplicons in cbPCR using wild type DNA and discrimination of mutant gDNA. (C) Using the optimized primers, completely mutant gDNA was discriminated from wild type or heterozygous mutant gDNA. Bands with expected sizes are marked with dots. The other bands were ignored as being either nonspecific or heteroduplexes. The calculated ratios between out- and in-amplicons are written below each lane. See S1 Fig for quantification of signals. WT or W: wild type, H: heterozygous, KO or K: knockout (mutant).

If we could overcome the problem of Plpp2 mutant detection, we should be able to determine how a failed primer design could be improved. We first did conventional PCR to test whether the F-in primer could bind to the template or not. When used without competition, the F-in and R-out primer set enabled the amplification of a detectable in-amplicon (Fig 3A). However, in cbPCR settings, the out-amplicon outcompeted the in-amplicon even in wild type DNA. This result shows that by doing PCR with individual primers, we can define whether the amplification of in-amplicon just failed or whether there was a too strong competition. If the inner primer does not work, we should either use the one with the other direction (R-in primer), or change PCR conditions (annealing temperature and/or enzyme) to make it work. If the inner primer does work, then adjustment of the balance of PCR amplification efficiency could be done. Since the inner primer did work for Plpp2, we did the latter. We tested F-out primers of different lengths, and found that slightly shorter ones (17 or 18 bp) enabled the detection of in-amplicons in cbPCR using wild type, but not mutant DNA (Fig 3B). Therefore, by refining primer design in failed experiments, we could design primer sets that discriminate mutants. As far as we tested, whenever an in-amplicon is efficiently detected by cbPCR using wild type gDNA, mutants are discriminated (when using the default primer design).

Since heterozygous mutant cells should also be present in screening experiments, we tested whether completely mutant cells can be correctly discriminated from them. For this experiment, we selected targets for which we had weak signals of in-amplicons when doing cbPCR (Figs 2D and 3B), because we thought that these targets might be more difficult to discriminate. We mixed 1:1 wild type and mutant DNA to obtain artificial heterozygous mutant DNA, and did cbPCR. Since we pre-selected targets with weak in-amplicon signals, some bands were difficult to visualize by eye, but a complete absence of in-amplicon was seen only when completely mutant DNA was used (Fig 3C). The difference was clearer when we analysed the signals from gel images by Image J (S1 Fig). The latter analysis revealed that the signals of in-amplicons tend to be reduced in heterozygous mutants, but less obviously than in completely mutant clones. Thus, the calculated ratios between out- and in-amplicons for heterozygotes were higher than for wild type, but the differences were not as pronounced as when using knockouts. This shows that the increase of amplicon ratios is not linear according to wild type copy number, and that a change in wild type copy number from one to zero causes a bigger shift than a change from two to one. Therefore, the results suggest that this strategy can be used for an easy screening to discriminate completely mutant clones from wild type or partially mutant clones.

### Testing the efficiency of mutant detection

**Fig 4:**
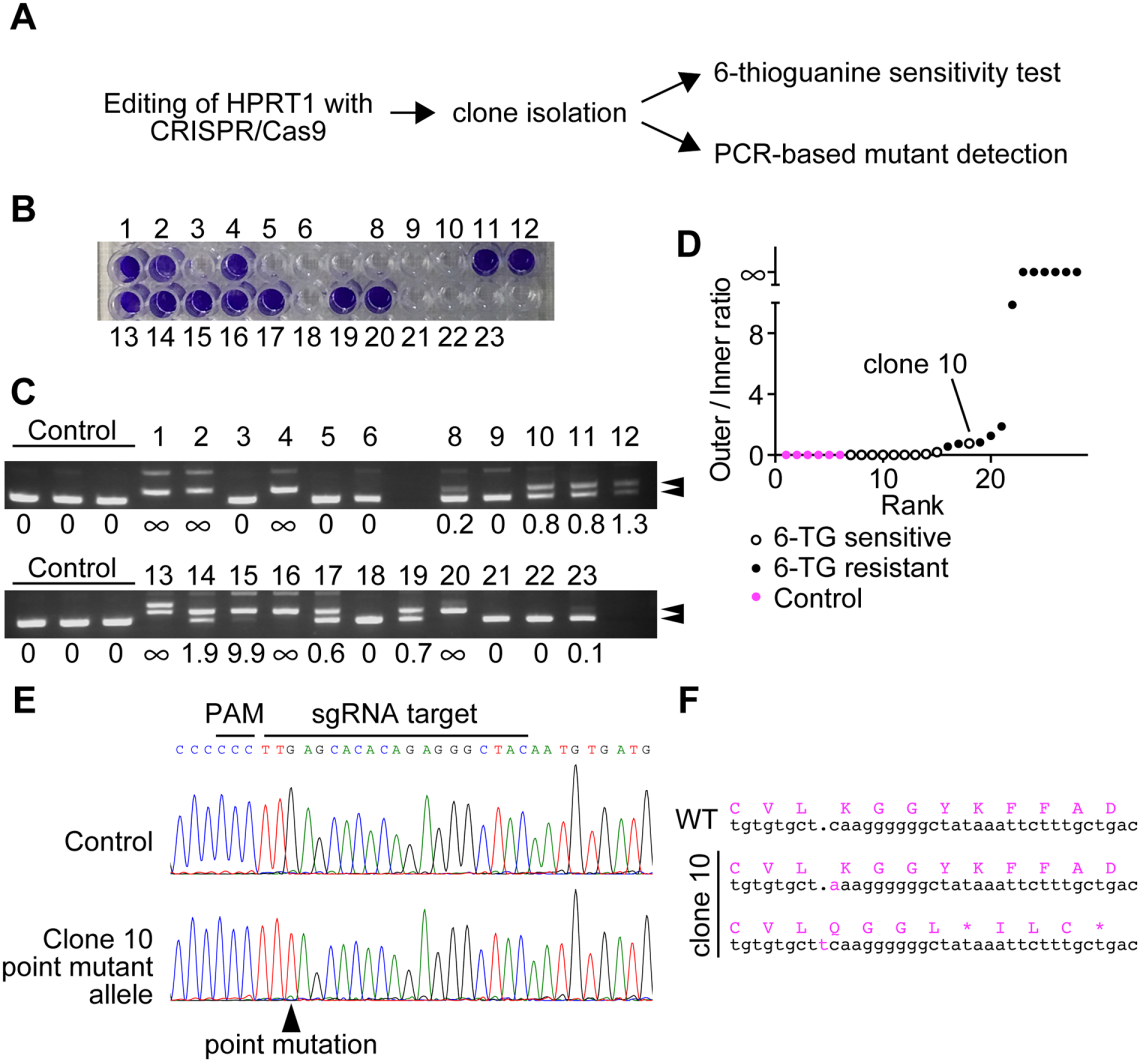
Accuracy of cbPCR to detect mutants. (A) Strategy to compare the efficacy of mutant detection by cbPCR. (B) Survival of different clones after HPRT1 editing and 6-TG selection. Viable cells were stained with crystal violet. Clone 7 is absent due to a growth arrest before experiments. (C) Result of cbPCR in the clones of (B). The bands of expected sizes are illustrated by arrowheads. (D) Bands of (C) were quantified and ratios between out- and in-amplicons were calculated. Ranking based on the calculated ratios discriminated 6-TG resistant clones, except for the 6-TG sensitive clone #10. (E) Clone 10 had a point mutation. (F) Sanger sequencing of clone 10 revealed that the point mutation was silent. The other allele had a +1 insertion. Mutations are illustrated in magenta.

Finally, we evaluated the error rate of cbPCR for mutant clone screening. For this, we mutated the HPRT1 gene in HeLa MZ cells using CRISPR/Cas9 and isolated clones. HPRT1-deficient cells are resistant to the toxicity of a nucleotide analog, 6-thioguanine (6-TG) [14]. Thus, by comparing 6-TG resistance and cbPCR results, we were able to evaluate how efficiently this method discriminates mutants from the others (Fig 4A). This experiment was done using two different guide RNAs targeting different sites for HPRT1, and samples from one experiment served as negative controls for the other experiment (since the mutation sites were distant enough). In one experiment, all 6-TG resistant clones (S2A Fig) had a complete absence of in-amplicons when analysed with cbPCR (S2B Fig). When out- to in-amplicon ratios were calculated and ranked, only 6-TG resistant clones had infinite values due to absent in-amplicons (S2C Fig). One 6-TG sensitive clone (clone 19) had an out- to in-amplicon ratio which was intermediate between controls and 6-TG resistant clones (S2 Fig). Sequencing analysis showed that this clone had a 2 bp deletion and a wild type allele. In addition, we selected six 6-TG sensitive clones that had ratios comparable to controls for sequencing, and found that all were completely wild type. Thus, for this target, the accuracy of mutant discrimination was 100%, and sequencing also suggested that partial mutants could be detected to some degree.

When we did the same experiment with another target site of HPRT1, some 6-TG resistant mutants (Fig 4B) had detectable in-amplicons (Fig 4C), but calculation of the out- to in-amplicon ratio enabled us to correctly discriminate them from the 6-TG sensitive clones (Fig 4D). This shows that mutants are not always detected as a complete lack of in-amplicon, but can be discriminated using the ratios between out- and in-amplicons. One exception was a 6-TG sensitive clone (#10) (Fig 4B) that was ranked as a mutant based on quantification of amplicon ratios (Fig 4C and 4D). By doing cbPCR with a shorter F-in primer, we could obtain a complete absence of in-amplicon in all the mutants (S3A Fig). However, the 6-TG sensitive clone #10 was still classified as a mutant even with this optimized primer design (S3B Fig). From this result, we speculated that clone #10 was indeed a mutant, but retained an active HPRT enzyme. We did sequencing to detect the pattern of mutations in clone #10, and found that one allele contained a point mutation without indels (Fig 4E). This mutation was silent, leading to a protein with wild type primary sequence (Fig 4F). Therefore, clone #10 was indeed a mutant, but was 6-TG sensitive due to a wild type HPRT protein. We further performed sequencing of all the clones having intermediate out- to in-amplicon ratios (between zero and infinite), as well as four clones each from samples having either zero or infinite ratios (Table 1). All the analysed clones with zero values were complete wild type. Heterozygous clones (8 and 23) had slightly increased ratios but at a lower degree than for homozygous mutant clones. As a tendency, mutants with bigger indels had bigger changes in ratios, and all mutants with infinite ratios had only large (>4) indels. Importantly, homozygous mutants with the smallest indels had bigger changes in ratios than the heterozygous mutant clone 23 that had a large deletion, meaning that the size of indels affect less the results than the wild type copy number. As a conclusion, cbPCR had also 100% accuracy (at least for the 6-TG resistant clones and the sequenced clones) for discriminating mutants of this locus, even without the further optimization (S3 Fig) that we did to obtain a complete disappearance of in-amplicons when doing cbPCR with mutant gDNA. This also shows that cbPCR is sensitive enough to detect even a point mutant. The sequencing results suggested that although the size of indels affects the quantification results, the presence or absence of wild type alleles have a bigger impact, thus enabling the discrimination of complete mutants.

**Table 1:** Comparison of mutation type and and cbPCR results.

Out / In ratio	Clone no.	6-TG sensitivity	Indel size
0	3	Sensitive	0
0	5	Sensitive	0
0	6	Sensitive	0
0	9	Sensitive	0
0.065	23	Sensitive	0, Del(134)
0.189	8	Sensitive	0, Ins(1)
0.556	17	Resistant	Ins(1)
0.739	19	Resistant	Ins(1)
0.769	10	Sensitive	0*, Ins(1)
0.831	11	Resistant	Ins(1)
1.265	12	Resistant	Del(32), Ins(1)
1.880	14	Resistant	Ins(1), Ins(2)
9.871	15	Resistant	Ins(1), Ins(5)
Infinite	1	Resistant	Del(30)
Infinite	2	Resistant	Del(9)
Infinite	4	Resistant	Del(5)
Infinite	13	Resistant	Del(11), Ins(>50)

Ins, insertion; Del, deletion; numbers in parentheses are the indel sizes.

* Point mutation

## Discussion

In this manuscript, we described competition-based PCR (cbPCR) as a method to detect mutant clones after genome editing experiments. The competitive factor in this experiment is very important. If the discrimination was based only on the lack of binding between a primer and its target, the primer (and the annealing temperature) should be designed to give a marginal binding to the target, which is lost even by the smallest indel. When competition is present, this strict optimum is no longer needed, since a decreased affinity is sufficient to give a different outcome. The advantage of this method is that it requires only equipment for PCR and agarose gel electrophoresis (or any other alternatives), thus can be performed in almost all laboratories, and at low cost. The method is so simple that we speculate that similar methods might already be used, even without recognizing that the detection is based on competition between amplicons, and not solely based on loss of inner primer binding. However, existing reviews [20] or online experimental guides (http://blog.addgene.org/crispr-101-validating-your-genome-edit) did not describe such a method for clone identification, thus we thought that it would be useful to share the theory and design optimization steps for cbPCR. We provide a protocol (S1 Protocol) to explain the steps for establishing cbPCR for different targets. Although optimization of additional factors such as extension time or cycling numbers (to test whether we need to reach an amplification plateau or not for efficient mutant detection) might be done, these issues were not investigated in this manuscript since the detection efficacy was already satisfactory with the current design.

The present method can discriminate homozygous mutants from the other undesired clones, even when the mutation is the same in both alleles. This is extremely important, since mutations induced by CRISPR/Cas9 experiments are not completely random [19]. Indeed, we have found cases where ~60% of the mutants had the same indel at the target site (S4 Fig). In such biased cases, many of the mutant clones will have the same mutation in all alleles, and screening of mutant clones with mismatch cleavage assays or heteroduplex mobility assay will result in the misidentification of many mutant clones, unless wild type PCR amplicon is added to all samples. Therefore, cbPCR is easier and might have a higher detection power than these other methods.

The success of mutant detection relies on the design of primers that can identify efficiently the mutants. The default setting in our laboratory (see Results and S1 Protocol) for primer design was good enough to detect mutants of 7 out of 8 genes. Furthermore, we could easily optimize primer design for the remaining one. Therefore, it is not difficult to obtain primer sets that work efficiently. One constraint for primer design is that the inner primer should overlap with the Cas9 cleavage site. It can be imagined that for some genes, this constraint will lead to the design of primers that are non-specific or that do not bind to the target, due to secondary structure or other reasons. We speculate that the non-specificity issue is not a problem, since we usually design guide RNAs that avoid off-target effects, thus the target should be unique in the genome. For the second issue, as far as we have tested, we did not find an inner primer that could not bind to its target. We routinely use two polymerases (ExTaq or PrimeSTAR GXL) for PCR, and all of the targets have been amplified by at least one of them. The freedom to design two orientations of the inner primer (although F-in orientation is preferable than the other) also reduces the probability to fail in the design of inner primers. In this manuscript, we often used mutant DNA that was generated a priori. From these experiments, we found that whenever the primers are designed based on the default setting and two bands are seen in cbPCR using wild type DNA, mutants can be discriminated. Therefore, even if one does not have mutant DNA a priori (as should be in most cases), a successful primer set could be designed based on cbPCR results using wild type DNA. If the in-amplicon is seen in wild type (and preferably also the out-amplicon, which should demonstrate that it is not overcompeted by the in-amplicon), then the primers should work. It should be noted that even if the primer is not fully optimized, the power of mutant detection by cbPCR is still very high (compare Fig 4 and S3 Fig).

In this manuscript, only random mutations induced by the widely-used wild type *Streptococcus pyogenes* Cas9 nuclease were analysed. However other genome editing methods exist to induce random mutations, such as Transcription Activator-Like Effector Nucleases, double-nickase using single-mutant Cas9, or CRISPR systems from other species [21]. We believe that cbPCR can detect mutations induced by other methods, as far as the mutation site is predictable. Since some of these genome editing strategies can induce staggered (instead of blunt) double strand breaks, it is possible that in such cases the prediction of where indels happen is more difficult, thus the placement of the inner primer might be more complicated. Indeed, indels generated by a double-nickase strategy could happen in two different sites separated from each other by more than 20 bp [5], making the design of an inner primer that can overlap with all the mutations impossible. Therefore, the type of double strand break should be considered before designing a cbPCR approach to analyse clones. Another application of genome editing is precise editing based on homology-directed repair using donor DNA [21]. We speculate that for such experiments, we can detect mutant clones with the desired mutation by designing an inner primer having a perfect match with the desired mutant site (and not the wild type site). By doing so, correctly mutated DNA should be detected by an increase in the ratio between in- and out-amplicons, since only correct mutants have efficient amplification of the in-amplicon, which is the inverse case of what is done in this manuscript. This hypothesis still has to be examined.

It should be emphasized that the experiment was specifically designed for detection of fully mutated clones. We do not expect this method to be quantitative enough for estimation of mutation rate in polyclonal cells, or for the detection of heterozygous mutant cells, although the results still suggest some degree of quantitation. Indeed, heterozygous mutants could be discriminated by cbPCR at least in all the experiments done in this manuscript, but only a limited number of targets were analysed until now. It should also be noted that some variability in signal ratios is seen between experiments (for example, compare Fig 1E and 1F), so it is appropriate to include at least one wild type sample in all experiments as a control, while avoiding comparison of ratio values between experiments done in different days. In addition, we recommend doing sequencing after the screening by cbPCR. Although the experiment with HPRT1 mutants suggests a very low misidentification rate, we have not yet evaluated the misidentification rate in large numbers of target genes. The ease of cbPCR is especially useful when large numbers of clones have to be analysed. Such a requirement might arise when multiplex genome editing is done. In such experiments, one can do cbPCR for all targets first, rank clones based on the ratio of out- and in-amplicons of each target, and sequence only genomic DNA of those cells that are expected to have mutations in all the targets. This will reduce drastically the time and cost needed to obtain the correct clones needed. We could obtain triple knockout cells easily using this strategy (S5 Fig). We do not recommend to directly sequence cbPCR products, since the in-amplicon might serve as a megaprimer for the out-amplicon, leading to sequencing reads that are partially wild type, even in completely mutated clones (S6 Fig).

To conclude, we established a method to discriminate fully mutated clones from the other undesired ones, based on a simple PCR experiment. The method is cost- and time effective, can be performed with standard equipment while being at least equally sensitive to existing methods.

## Supporting information

S1 Fig Analysis of gel images. (PDF)

S2 Fig Evaluation of the accuracy of cbPCR. (PDF)

S3 Fig Further optimization of primers for detecting HPRT1 mutant clones. (PDF)

S4 Fig Example of biased indels. (PDF)

S5 Fig Isolation of triple mutants with cbPCR. (PDF)

S6 Fig Artefact of sequencing when doing direct sequencing from cbPCR. (PDF)

S1 Protocol Supplementary protocol to design cbPCR experiments. (DOCX)

S1 Table Primers used for standard PCR. (DOCX)

S2 Table Target sequences for CRISPR/Cas9 editing in the indicated cell lines. (DOCX)

S3 Table Primers used for optimization and application of cbPCR. (DOCX)

S4 Table Quantification of out- to in-amplicon ratios from cbPCR results in S5 Fig. (DOCX)
