# Supplementary material for: Detection of genome-edited mutant clones by a simple competition-based PCR method
Source: PLoS One. 2017 Jun 6;12(6):e0179165. doi: 10.1371/journal.pone.0179165 (PMC5460891; doi:10.1371/journal.pone.0179165)
Supplement: S1 Fig — (PDF) [file pone.0179165.s001.pdf]

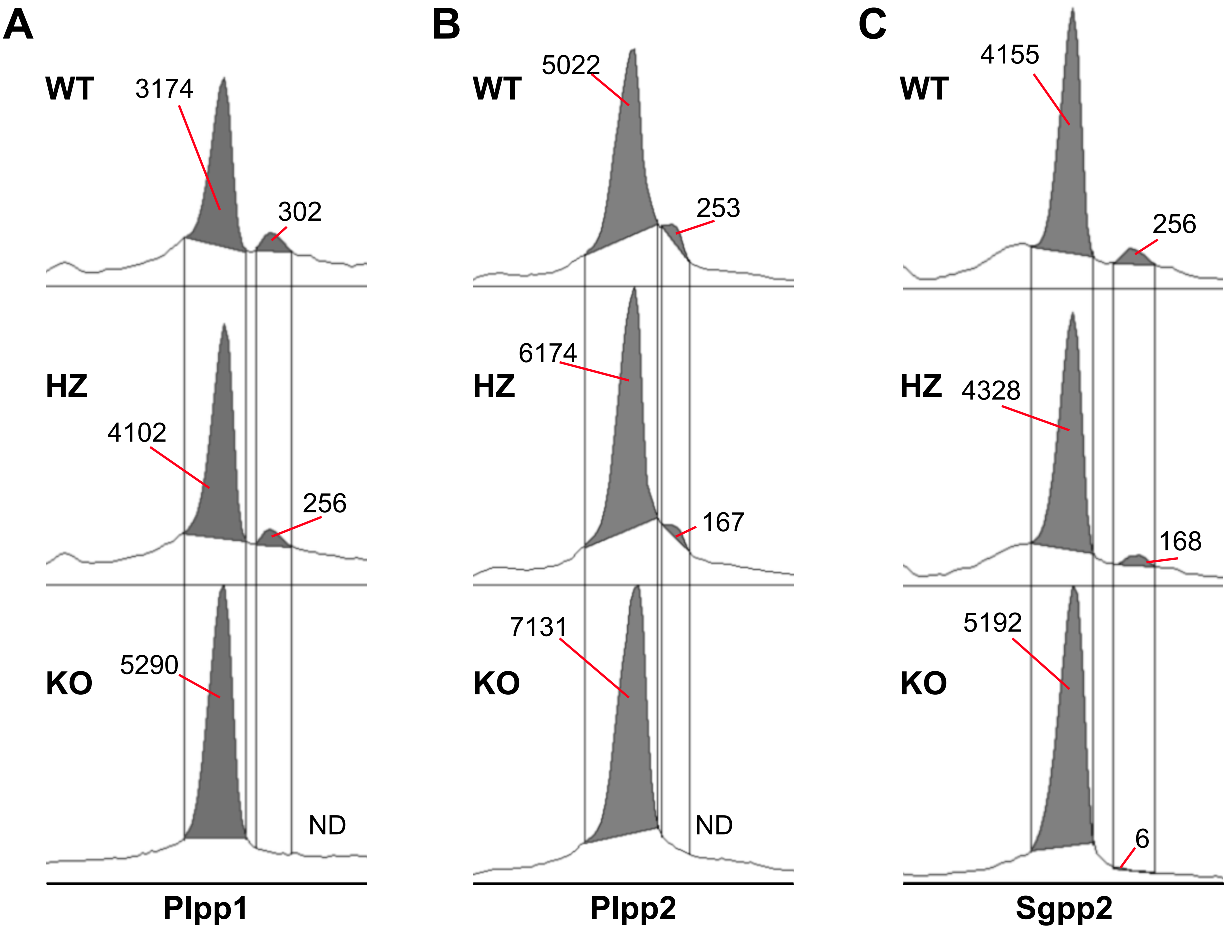

S1 Fig

Analysis of gel images. (A-C) To quantify the density of PCR bands from Fig 3C, plots of signal intensities were obtained using ImageJ. Areas under the curve (in gray) of peaks having the same migration were measured and compared accross genotypes. In these plots, the direction of band migration is rightward. WT: wild type, HZ: heterozygous mutant, KO: knockout (mutant).
