# Supplementary material for: Detection of genome-edited mutant clones by a simple competition-based PCR method
Source: PLoS One. 2017 Jun 6;12(6):e0179165. doi: 10.1371/journal.pone.0179165 (PMC5460891; doi:10.1371/journal.pone.0179165)
Supplement: S2 Fig — (PDF) [file pone.0179165.s002.pdf]

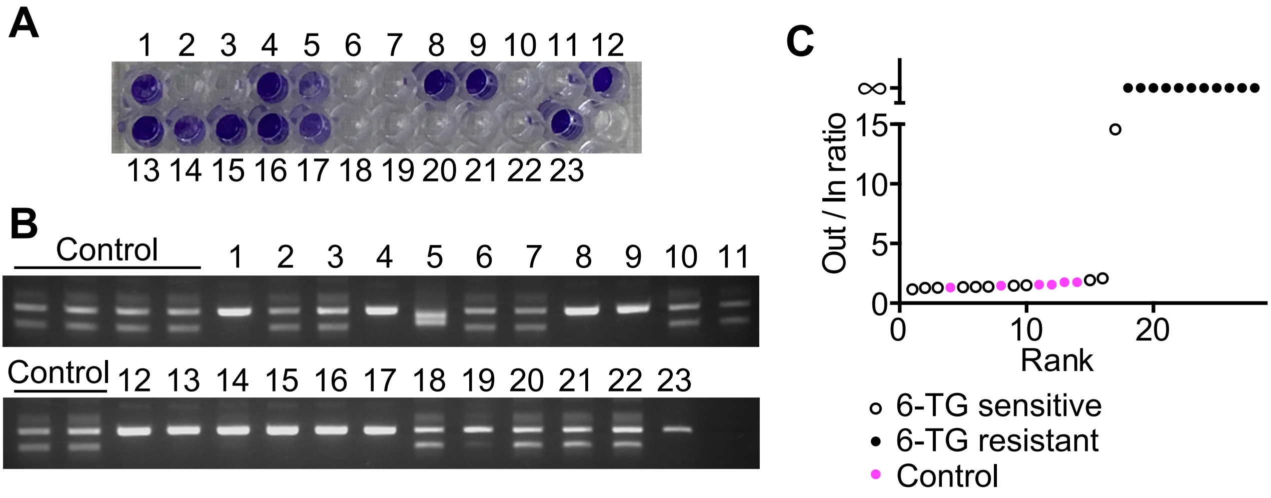

S2 Fig

Evaluation of the accuracy of cbPCR. (A) Survival of different clones after HPRT1 editing at a site different from Fig 4. Viable cells are stained with crystal violet. (B) Result of cbPCR in the clones of (A). (C) All the 6-TG resistant clones have absent in-amplicons and are discriminated from the others.
