# Supplementary material for: Detection of genome-edited mutant clones by a simple competition-based PCR method
Source: PLoS One. 2017 Jun 6;12(6):e0179165. doi: 10.1371/journal.pone.0179165 (PMC5460891; doi:10.1371/journal.pone.0179165)
Supplement: S3 Fig — (PDF) [file pone.0179165.s003.pdf]

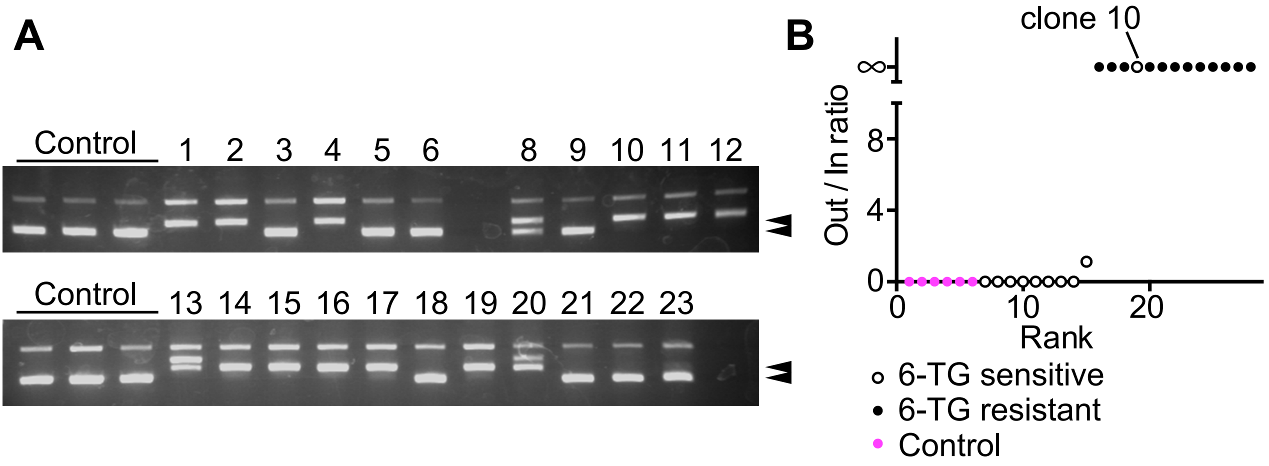

S3 Fig

Further optimization of primers for detecting HPRT1 mutant clones. (A) Result of cbPCR using an F-in primer shorter than in Fig 4. The bands of expected sizes are illustrated by arrowheads. (B) Quantification of band intensities reveals a total lack of in-amplicon in 6-TG resistant clones as well as clone #10.
