# Supplementary material for: Detection of genome-edited mutant clones by a simple competition-based PCR method
Source: PLoS One. 2017 Jun 6;12(6):e0179165. doi: 10.1371/journal.pone.0179165 (PMC5460891; doi:10.1371/journal.pone.0179165)
Supplement: S4 Fig — (PDF) [file pone.0179165.s004.pdf]

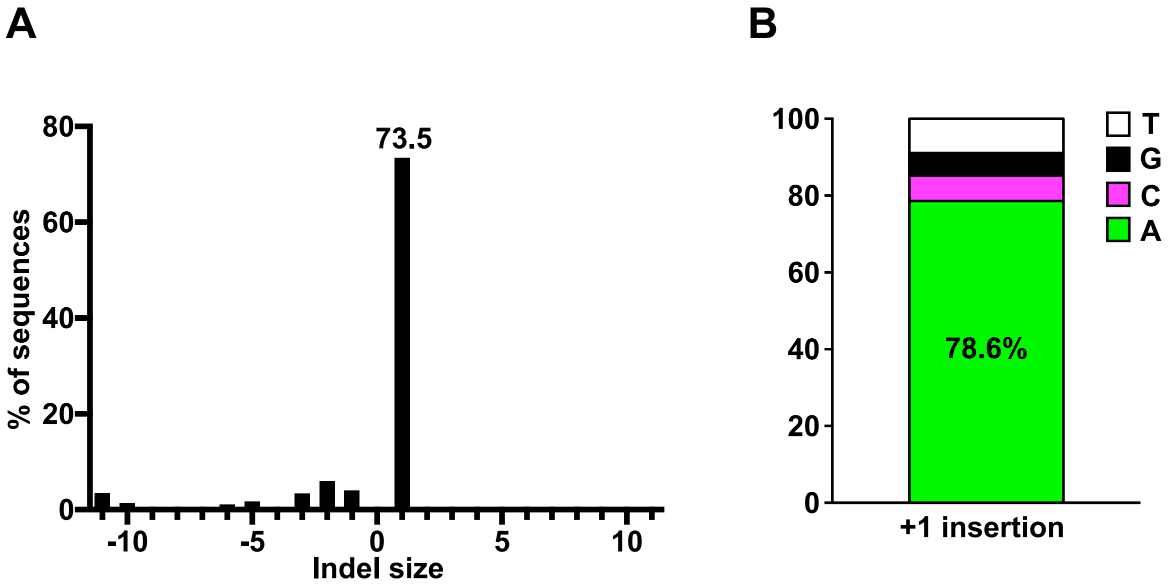

S4 Fig

Example of biased indels. HPRT1 locus of HeLa MZ cells was edited using the same constructs as Fig 4C, but this time polyclonal mutant cells were obtained by doing 6-TG selection in bulk cells, without clone isolation, generating a mixture of cells with various mutations. Indels in the HPRT1 locus were analyzed by TIDE analysis (see Materials and Methods). (A) Despite the polyclonality of the cells, 73.5% of the annotable alleles had a +1 insertion. (B) Among the +1 indels, 78.6% had an insertion of A. Thus, genome editing at this locus results in biased indel formation, with 58% of the alleles having the same +1 adenine insertion.
