# Supplementary material for: Detection of genome-edited mutant clones by a simple competition-based PCR method
Source: PLoS One. 2017 Jun 6;12(6):e0179165. doi: 10.1371/journal.pone.0179165 (PMC5460891; doi:10.1371/journal.pone.0179165)
Supplement: S5 Fig — (PDF) [file pone.0179165.s005.pdf]

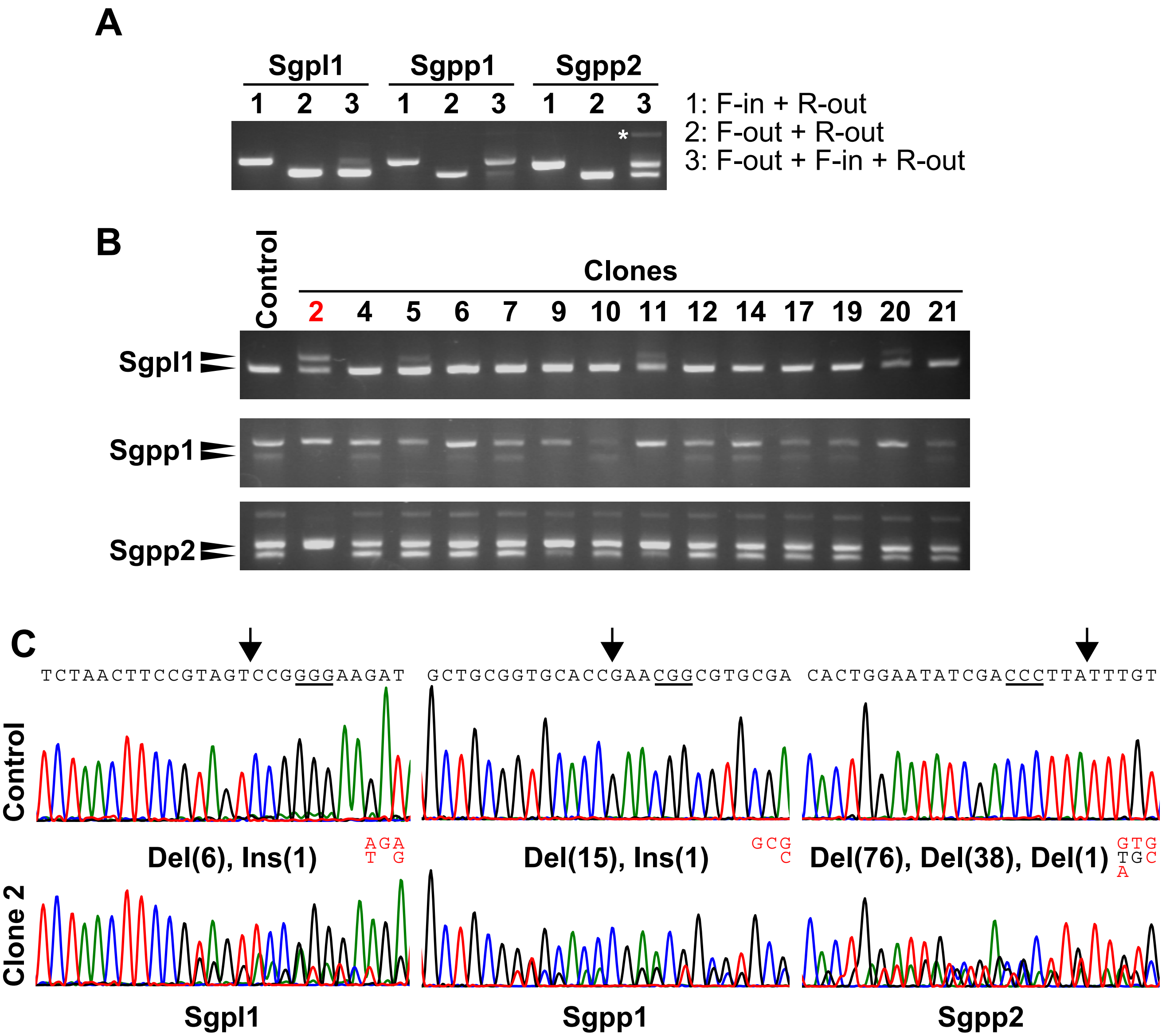

S5 Fig

Isolation of triple mutants with cbPCR. Using cbPCR, McA-RH7777 clones having mutations in both Sgpl1, Sgpp1, and Sgpp2 loci (different from Fig 2D) were isolated. (A) PCR primers for cbPCR of each locus were designed and successful amplification was confirmed by doing PCR with the indicated primer pairs. A nonspecific or heteroduplex band is illustrated by an asterisk, which was ignored for analysis. (B) Result of cbPCR analysis of different loci and different clones. Quantification is shown in S4 Table. (C) Clone 2, which had the biggest changes in cbPCR outcomes, was analyzed by Sanger sequencing. The same regions of sequencing data are aligned from a wild type control and clone 2, and the absence of wild type signals can be clearly seen in the rightmost peaks. Arrows illustrate the cleavage site, and PAM sequences are underlined. The annotations of indels analyzed by TIDE or manually are written above the spectra. Del: deletion, Ins: insertion, the numbers in parentheses are indel sizes.
