# Supplementary material for: Detection of genome-edited mutant clones by a simple competition-based PCR method
Source: PLoS One. 2017 Jun 6;12(6):e0179165. doi: 10.1371/journal.pone.0179165 (PMC5460891; doi:10.1371/journal.pone.0179165)
Supplement: S6 Fig — (PDF) [file pone.0179165.s006.pdf]

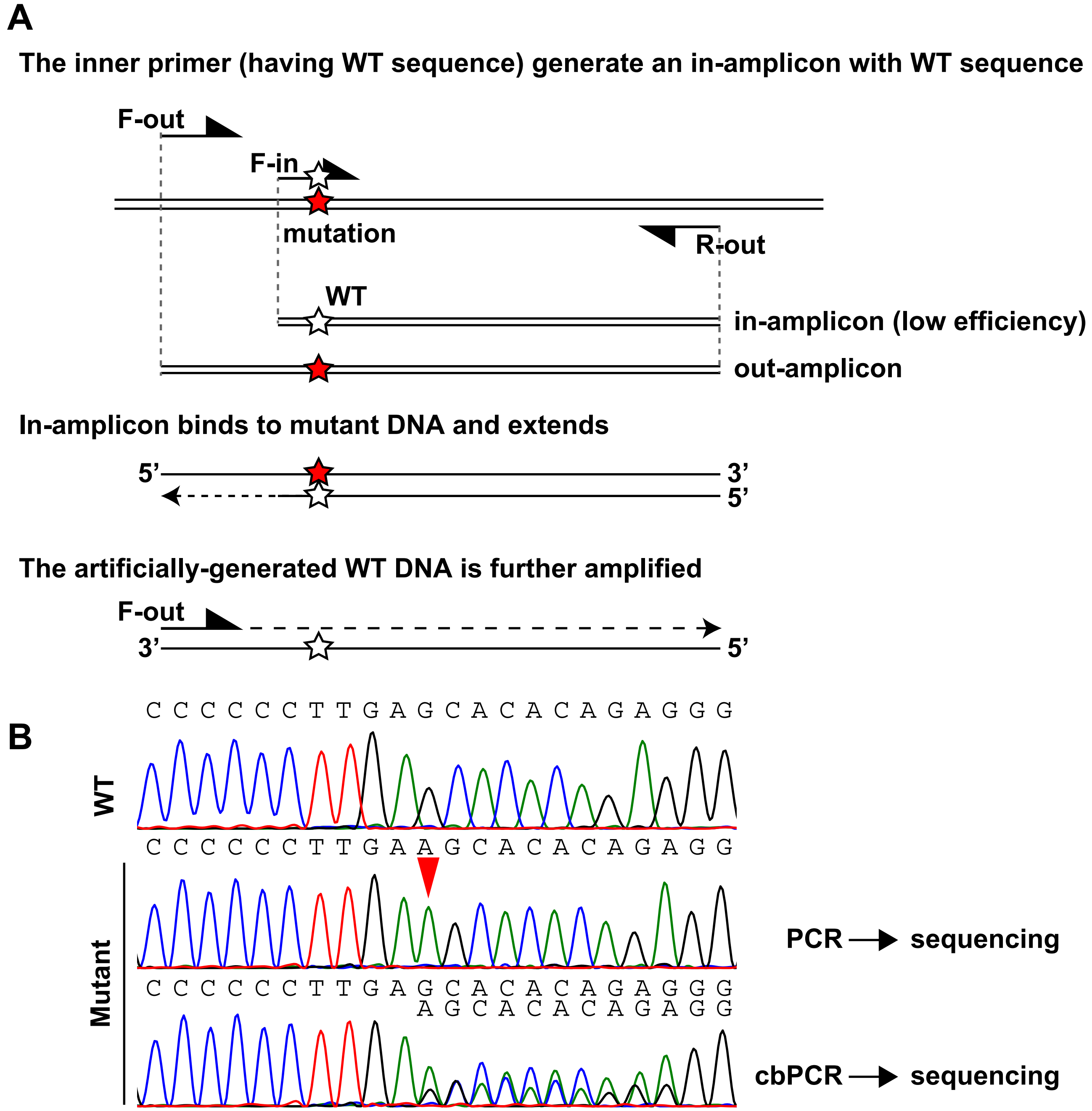

S6 Fig

Artifact of sequencing when doing direct sequencing from cbPCR. (A) Explanation of the process in which a wild type amplicon can be generated even when the target is fully mutated. The small fraction of in-amplicon that is generated by priming of F-in in the mutant site generates a in-amplicon with wild type sequence. This fragment can then work as a megaprimer and generate a wild type out-amplicon. (B) Sequencing results of an HPRT1-mutant HeLa MZ clone having a +1 insertion (arrowhead) after conventional PCR or cbPCR. Although exactly the same DNA samples were used, direct sequencing after cbPCR resulted in the detection of wild type signals, which was an artifact. The same wild type sequence as Fig 4E is shown for comparison.
