# Supplementary material for: Detection of genome-edited mutant clones by a simple competition-based PCR method
Source: PLoS One. 2017 Jun 6;12(6):e0179165. doi: 10.1371/journal.pone.0179165 (PMC5460891; doi:10.1371/journal.pone.0179165)
Supplement: S1 Protocol — (DOCX) [file pone.0179165.s007.docx]

**Supplementary Protocol**

Initial design of outer primers

Since the optimal direction of the inner primer depends on the location of the mutation in the out-amplicon, we first design the outer primers. Using the sequence of the guide RNA, we do BLAT analysis (http://genome.ucsc.edu/cgi-bin/hgBlat?command=start) (1) to obtain the corresponding genome sequence, starting from 300 bp upstream to 600 bp (total 920 bp) downstream of the guide RNA target and use it as a query for primer BLAST (https://www.ncbi.nlm.nih.gov/tools/primer-blast/) to design primers. The forward and reverse primers are designed to be located between positions 1-150 and 771-920, respectively. If this design does not give good primers, we repeat it using genome region of the other strand (by obtaining 600 bp upstream to 300 bp downstream of the guide RNA using BLAT). In our experience, even if primer BLAST suggests the possibility of non-specific amplification, the primers are often specific enough. To test whether these primers are working, PCR reactions are carried out using Ex Taq or PrimeSTAR GXL (TAKARA Clonetech) as follows:

for Ex Taq:

Mixture

10x Ex Taq buffer 1 µl
dNTP mix (2.5 mM each) 0.8 µl

DMSO 0.5 µl

primers (10 µM each) 0.2 µl each

Ex Taq 0.05 µl

template 1 µl

water 6.25 µl

Reactions

1: 94ºC 5:00

2: 94ºC 0:30

3: 60ºC 0:30

4: 72ºC 0:50

Repeat 2-4 35 times

5: 72ºC 5:00

6: keep at 4ºC

for PrimeSTAR GXL:

Mixture

5x PrimeSTAR GXL buffer 2 µl
dNTP mix (2.5 mM each) 0.8 µl

primers (10 µM each) 0.2 µl each

PrimeSTAR GXL 0.2 µl

template 1 µl

water 5.6 µl

Reactions

1: 98ºC 0:10

2: 60ºC 0:15

3: 68ºC 0:50

Repeat 1-3 35 times

4: keep at 4ºC

PCR products are analysed by agarose gel electrophoresis, typically using 1.5% (w/v) gels in TBE buffer (89 mM Tris-borate, 2 mM EDTA). Bands are visualized using EZ-VISION DNA dye (amresco) or ethidium bromide (Sigma-Aldrich). If a single band at the estimated size is seen, we use this primer set as the initial outer primers. These primers can be used for TIDE analysis when one needs to analyze genome editing efficiency in bulk cells, e.g. before isolation of clones. If the primers do not work, we select new ones from the other candidates designed by primer BLAST. Although we might change the PCR conditions for each primer, we prefer to redesign primers, since if all the primers work at the same condition, more experiments can be done in parallel. We first test PCR with Ex Taq, but if multiple primer sets do not work, we test them with PrimeSTAR GXL.

Design of primers for cbPCR

We usually design the inner primer after confirming that the initial outer primers are working. The inner primer should be oriented in a way that it amplifies the larger fragment (the F-in orientation in Fig 2A). The estimated Cas9 cleavage site is between 3 and 4 bases upstream of the NGG proximal adjacent motif. We design the inner primer in order that it binds a region until 3 bases downstream of the cleavage site (see Fig 2A as an example). Then, we use primers with varying length (by changing the 5’ end) as an input for primer BLAST, and the longest one not exceeding a melting temperature (Tm) of 60ºC is selected as F-in primer. In our experience, primers shorter than 16 bases rarely worked. Therefore, if the above design gives rise to a primer shorter than 16 bases, we simply use a 16mer, irrespectively of its Tm. Next, we redesign the F-out primer similarly, adjusting its length to give it a Tm lower than that of F-in (although we routinely do this, we did not test extensively whether this redesign is really beneficial). Using the same PCR conditions as above, except that 0.2 µl of water is replaced by 0.2 µl of the F-in primer (10 µM), we test whether the primer set works using DNA from wild type cells. If one has already a mutant DNA at this point, this can also be used at this point to confirm that mutants are discriminated. If the in-amplicon is detected with sufficient signal in cbPCR using wild type DNA, the primers are expected to be working for the discrimination of mutant DNA. Although this is not a requisite, it is preferable that the out-amplicon is also detected, since we can expect a good amplification of this one when using mutant DNA.

Optional refinement of primers

When the in-amplicon is not seen in cbPCR using wild type DNA, the primer design is a failure (see Plpp2 lane in Fig 2D). This might happen when the F-in primer is not binding to its target, or when the out-amplicon outcompetes the in-amplicon. To discriminate the possibilities, one can do PCR with individual pairs of primers (F-out + R-out, F-in + R-out, or the three mixed) like in Fig 3A. If the F-in + R-out pair does not give any band, the F-in primer is not working (since the R-out primer is pre-validated). In this case, one can either change the length of F-in, or use the less optimal inner primer orientation (R-in), or use other PCR enzymes. Among the above strategies, changing the length is the least advisable, since longer primers are more tolerant to mutations (Fig 2C). When the out-amplicon is outcompeting the in-amplicon, one can test shorter F-out primers to see which length enables the detection of in-amplicons in cbPCR (Fig 3B). When the out-amplicon is outcompeted by the in-amplicon when doing cbPCR with wild type DNA, it is possible that the in-amplicon is not completely disappeared when using mutant DNA (clones 10, 11, 12, 14, 15, 17, and 19 in Fig 4C). Although this is not a problem since we can calculate the out- to in-amplicon ratio, one might prefer to detect mutants as a total disappearance of in-amplicon. Since we use pre-validated outer primers, the reason that the out-amplicon is not seen is likely to be due to a too strong amplification of in-amplicon when using wild type. Therefore, one can design shorter F-in primers to test which length will lead to a detectable out-amplicon in cbPCR using wild type DNA, as is done in S3 Fig.

**Reference**

1. Kent, W.J. (2002) BLAT--the BLAST-like alignment tool. *Genome Res*, **12**, 656-664.
