# Supplementary material for: Detection of genome-edited mutant clones by a simple competition-based PCR method
Source: PLoS One. 2017 Jun 6;12(6):e0179165. doi: 10.1371/journal.pone.0179165 (PMC5460891; doi:10.1371/journal.pone.0179165)
Supplement: S1 Table — (DOCX) [file pone.0179165.s008.docx]

**S1 Table**

| Primer | Sequence | Enzyme |
| --- | --- | --- |
| Amplification of the sgRNA expression cassette from pX330 for pUC-U6-sg construction  (the overlap with pUC19 backbone is illustrated in bold letters) | | |
| Forward | ATGATTACGCCAAGCTTgagggcctatttcccatgattc | PrimeSTAR GXL |
| Reverse | GACGGCCAGTGAATTCgcgctaaaaacggactagcc |  |
| Amplification of the plasmid backbone pUC19 | | |
| Forward | GAATTCACTGGCCGTCGTTT | PrimeSTAR GXL |
| Reverse | AAGCTTGGCGTAATCATGGTC |  |
| Amplification of genomic loci for TIDE analysis | | |
| Plpp1-F | CGAGTCCAAACTGCCCAGG | Ex Taq |
| Plpp1-R | AGCTTCTTCAGACCTTGTCGG |  |
| Plpp2-F | CCTGGCCTCGGTGACATTAG | Ex Taq |
| Plpp2-R | GGGGAGTCACAGTGCTTGAA |  |
| Plpp3-F | GACTAGCGAACAGTTTGGGGT | PrimeSTAR GXL |
| Plpp3-R | ACCTGGGTAGAGCCACGTTC |  |
| Sgpl1-F | AGATACACCAAAATGCTGACACT | Ex Taq |
| Sgpl1-R | ACTCAGGATGCTAGCCGTTC |  |
| Sgpp1-F | AACTCGTTGACGGGCGAG | Ex Taq |
| Sgpp1-R | ACCGGGTCTCAGTTACCAAG |  |
| Sgpp2-F | TGCAGTCACAGGTTGGTCTG | Ex Taq |
| Sgpp2-R | GGAATCCATGTGGTACTGGCT |  |
| Sphk1-F | TACCTCGTGCATCAGACCGT | Ex Taq |
| Sphk1-R | AAGAGGTATTGCGGCGTCC |  |
| Sphk2-F | ATATTGTCAAGGGAGGGACAGAC | Ex Taq |
| Sphk2-R | TGCAACAGAGTTAAAGTGTCCT |  |
| Amplification of HPRT1 for cloning, sequencing, and TIDE analysis | | |
| HPRT1-F (Fig 4) | AGCAAGTATGGTTTGCAGAGA | PrimeSTAR GXL, Ex Taq |
| HPRT1-R (Fig 4) | ACAGGGTTCGCCATGTTACC |  |
| HPRT1-F (S2 Fig) | GCTGTCATTGATCCTGCACCT | Ex Taq |
| HPRT1-R (S2 Fig) | AGGAGGTGAGGCCGACTT |  |
| Amplification of genomic loci of triple knockout clones for TIDE analysis | | |
| Sgpl1-F2 | AGAAGATACACCAAAATGCTGACA | Ex Taq |
| Sgpl1-R2 | CACGGTCCTCTCATCAAAGCC |  |
| Sgpp1-F2 | GGCCGGATCAATTCCCGAGT | Ex Taq |
| Sgpp1-R2 | GGTATTTTGGACTGGAAGAGCCA |  |
| Sgpp2-F2 | CAGCACCCTGGTAGTGAGTT | Ex Taq |
| Sgpp2-R2 | AAAGGGTTGTTGGACCTCCC |  |

S1 Table. Primers used for standard PCR
