# Supplementary material for: Detection of genome-edited mutant clones by a simple competition-based PCR method
Source: PLoS One. 2017 Jun 6;12(6):e0179165. doi: 10.1371/journal.pone.0179165 (PMC5460891; doi:10.1371/journal.pone.0179165)
Supplement: S2 Table — (DOCX) [file pone.0179165.s009.docx]

**S2 Table**

| Gene | Target | PAM | Plasmid | Cells |
| --- | --- | --- | --- | --- |
| Plpp1 | gcaacacgcaaatcacatcg | agg | pX330 | McA-RH7777 |
| Plpp2 | ctatgtggctgccatctaca | agg | pX330 | McA-RH7777 |
| Plpp3 | gtccctgagagtaagaacgg | cgg | pX330 | McA-RH7777 |
| Sgpl1 | aggcttacggagaattcacg | tgg | pX330 | McA-RH7777 |
| Sgpl1#2 | cctctaacttccgtagtccg | ggg | pX330 | McA-RH7777 |
| Sgpp1 | aggacatcatccgttggccg | cgg | pX330 | McA-RH7777 |
| Sgpp1#2 | gactgctgcggtgcaccgaa | cgg | pX330 | McA-RH7777 |
| Sgpp2 | ataagggtcgatattccagt | ggg | pX330 | McA-RH7777 |
| Sgpp2#2 | ccaatcttctggacaaataa | ggg | pX330 | McA-RH7777 |
| Sphk1 | gccttgcccttgccaccccg | ggg | pX330 | McA-RH7777 |
| Sphk2 | ttatgtggagaatcgtgcag | agg | pX330 | McA-RH7777 |
| Hprt1 | cttttatgtcccccgttgac | tgg | pUC-U6-sg | McA-RH7777 |
| HPRT1 | gtagccctctgtgtgctcaa | ggg | pUC-U6-sg | HeLa MZ |
| HPRT1 | tggattatactgcctgacca | agg | pUC-U6-sg | HeLa MZ |

S2 Table. Target sequences for CRISPR/Cas9 editing in the indicated cell lines
