# Supplementary material for: Detection of genome-edited mutant clones by a simple competition-based PCR method
Source: PLoS One. 2017 Jun 6;12(6):e0179165. doi: 10.1371/journal.pone.0179165 (PMC5460891; doi:10.1371/journal.pone.0179165)
Supplement: S3 Table — (DOCX) [file pone.0179165.s010.docx]

**S3 Table**

| Gene | Primer | Sequence | Tm | Enzyme | Figures |
| --- | --- | --- | --- | --- | --- |
| Sgpl1 | F-out | AGATACACCAAAATGCTGACACT | 58.3 | Ex Taq | 1EF,2BCD |
|  | R-out | ACTCAGGATGCTAGCCGTTC | 59.5 |  | 1BEF,2BCD |
|  | F-in (26mer) | GGGTGCAGTGGATTGCTCCACGTGAA | 69.3 |  | 1BEF,2BC |
|  | F-in (23mer) | TGCAGTGGATTGCTCCACGTGAA | 65.0 |  | 2C |
|  | F-in (21mer) | CAGTGGATTGCTCCACGTGAA | 60.9 |  | 2C |
|  | F-in (20mer) | AGTGGATTGCTCCACGTGAA | 59.6 |  | 2D |
|  | F-in (18mer) | TGGATTGCTCCACGTGAA | 56.5 |  | 2C |
|  | R-in | TTTCTTTGCAGGCTTACGGAGAATTCACG | 66.0 |  | 2B |
| Sgpl1 #2 | F-out | CTAGAAGATACACCAAAATGCTGACA | 59.4 | Ex Taq | S5AB |
|  | R-out | CACGGTCCTCTCATCAAAGCC | 61.0 |  | S5AB |
|  | F-in | GCCTCTAACTTCCGTAGTCCG | 59.9 |  | S5AB |
| Plpp1 | F-out | GAGTCCAAACTGCCCAGG | 57.6 | Ex Taq | 2D,3C |
|  | R-out | AGCTTCTTCAGACCTTGTCGG | 60.0 |  | 2D,3C |
|  | F-in | CGTACGTGGTCCTCGATGT | 59.2 |  | 2D,3C |
| Plpp2 | F-out (19mer) | CTGGCCTCGGTGACATTAG | 57.6 | Ex Taq | 2D,3AB |
|  | F-out (18mer) | TGGCCTCGGTGACATTAG | 56.3 |  | 3B |
|  | F-out (17mer) | GGCCTCGGTGACATTAG | 54.1 |  | 3BC |
|  | F-out (16mer) | GCCTCGGTGACATTAG | 50.9 |  | 3B |
|  | R-out | GGGGAGTCACAGTGCTTGAA | 59.9 |  | 2D,3ABC |
|  | F-in | AAAGGTTCCCAGAACCTTGTAGA | 59.3 |  | 2D,3ABC |
| Plpp3 | F-out | ACTAGCGAACAGTTTGGGGT | 59.2 | Prime  STAR GXL | 2D |
|  | R-out | ACCTGGGTAGAGCCACGTTC | 61.5 |  | 2D |
|  | F-in | CGTCCCTGAGAGTAAGAACGG | 59.9 |  | 2D |
| Sgpp1 | F-out | CTCGTTGACGGGCGAG | 57.3 | Ex Taq | 2D |
|  | R-out | ACCGGGTCTCAGTTACCAAG | 59.0 |  | 2D |
|  | F-in | GACATCATCCGTTGGCCG | 58.6 |  | 2D |
| Sgpp1 #2 | F-out | GCCGGATCAATTCCCGAGT | 59.9 | Ex Taq | S5AB |
|  | R-out | GGTATTTTGGACTGGAAGAGCCA | 60.6 |  | S5AB |
|  | F-in | GCTGCGGTGCACCGAA | 60.7 |  | S5AB |
| Sgpp2 | F-out | GCAGTCACAGGTTGGTCTG | 58.4 | Ex Taq | 2D,3C |
|  | R-out | GGAATCCATGTGGTACTGGCT | 59.8 |  | 2D,3C |
|  | F-in | GACAAATAAGGGTCGATATTCCAGT | 58.7 |  | 2D,3C |
| Sgpp2 #2 | F-out | AGCACCCTGGTAGTGAGTT | 57.8 | Ex Taq | S5AB |
|  | R-out | AAAGGGTTGTTGGACCTCCC | 59.8 |  | S5AB |
|  | F-in | CCACTGGAATATCGACCCTTATTT | 58.4 |  | S5AB |
| Sphk1 | F-out | TACCTCGTGCATCAGACCGT | 61.0 | Ex Taq | 2BD |
|  | R-out | AAGAGGTATTGCGGCGTCC | 60.2 |  | 2BD |
|  | F-in (20mer) | GCCTTGCCCTTGCCACCCCG | 68.9 |  | 2B |
|  | F-in (16mer) | TGCCCTTGCCACCCCG | 62.2 |  | 2D |
|  | R-in | TGCTGCTGAACCCCCGGGGT | 68.7 |  | 2B |
| Sphk2 | F-out | ATATTGTCAAGGGAGGGACAGAC | 59.5 | Ex Taq | 2D |
|  | R-out | TGCAACAGAGTTAAAGTGTCCT | 57.9 |  | 2D |
|  | F-in | CGCTGGGCCTCTGCAC | 60.5 |  | 2D |
| HPRT1  #1 | F-out | AGCAAGTATGGTTTGCAGAGA | 57.6 | Ex Taq | 4C,S3A |
|  | R-out | ACAGGGTTCGCCATGTTACC | 60.3 |  | 4C,S3A |
|  | F-in (22mer) | GAATTTATAGCCCCCCTTGAGC | 58.8 |  | 4C |
|  | F-in (20mer) | ATTTATAGCCCCCCTTGAGC | 57.0 |  | S3A |
| HPRT1  #2 | F-out | CTGTCATTGATCCTGCACCT | 57.6 | Ex Taq | S2B |
|  | R-out | AGGAGGTGAGGCCGACTT | 59.9 |  | S2B |
|  | F-in | CAGACTTTGCTTTCCTTGGTCA | 59.1 |  | S2B |

S3 Table. Primers used for optimization and application of cbPCR.
