# Supplementary material for: Detection of genome-edited mutant clones by a simple competition-based PCR method
Source: PLoS One. 2017 Jun 6;12(6):e0179165. doi: 10.1371/journal.pone.0179165 (PMC5460891; doi:10.1371/journal.pone.0179165)
Supplement: S4 Table — (DOCX) [file pone.0179165.s011.docx]

**S4 Table**

|  | Sgpl1 | Sgpp1 | Sgpp2 |
| --- | --- | --- | --- |
| Control | 0.00 | 4.25 | 1.70 |
| 2 | 0.69 | ∞ | ∞ |
| 4 | 0.00 | 5.21 | 1.13 |
| 5 | 0.06 | ∞ | 1.25 |
| 6 | 0.00 | 29.41 | 1.58 |
| 7 | 0.00 | 3.38 | 1.44 |
| 9 | 0.00 | ∞ | 6.14 |
| 10 | 0.00 | 0.82 | 1.88 |
| 11 | 0.11 | ∞ | 6.15 |
| 12 | 0.00 | 6.43 | 1.43 |
| 14 | 0.00 | 4.47 | 1.50 |
| 17 | 0.00 | 2.98 | 1.57 |
| 19 | 0.00 | 6.30 | 1.54 |
| 20 | 0.14 | ∞ | 2.04 |
| 21 | 0.00 | 3.82 | 1.47 |

S4 Table. Quantification of out- to in-amplicon ratios from cbPCR results in S5 Fig.
